# The complete mitochondrial genome of *Amyda cartilaginea* (Testudines: Trionychidae)

**DOI:** 10.1080/23802359.2020.1832595

**Published:** 2020-11-03

**Authors:** Lin Cui, Dingqi Rao, Mingwang Zhang

**Affiliations:** a College of Animal Science and Technology, Sichuan Agricultural University, Chengdu, PR China; b Animal Genetic Resources Exploration and Innovation Key Laboratory of Sichuan Province, Sichuan Agricultural University, Chengdu, China; c State Key Laboratory of Genetic Resources and Evolution, Kunming Institute of Zoology, Chinese Academy of Sciences, Kunming, China

**Keywords:** *Amyda cartilaginea*, Trionychidae, mitogenome, next-generation sequencing, phylogenetic relationship

## Abstract

The Asiatic softshell turtle, also known as the black-rayed softshell turtle (*Amyda cartilaginea;* Accession no: MT039230), is found in northeastern India (Mizoram), Brunei Darussalam, Indonesia, Malaysia, Singapore, Myanmar, Laos, Vietnam, Cambodia, and Thailand. This turtle is thought to have been introduced into the Sunda Islands, Sulawesi, and Yunnan, China, through the Malay Peninsula to Sumatra, Java, and Borneo. Herein, we determined the complete mitochondrial genome of *A. cartilaginea* for the first time using next-generation sequencing (NGS). The assembled mitogenome was 16,763 bp in length and encoded 13 protein-coding genes (PCGs), 22 transfer RNA (tRNA) genes, two ribosomal RNA genes (12S rRNA and 16S rRNA), and one control region (CR). The PCGs based maximum-likelihood phylogeny discriminated *A. cartilaginea* from other Testudines and clusters within family Trionychidae with the sister taxa of *Nilssonia nigricans*.

Asiatic softshell turtle or black-rayed softshell turtle (*Amyda cartilaginea*), is widely distributed in northeastern India (Mizoram), Brunei Darussalam, Indonesia, Malaysia, Singapore, Myanmar, Laos, Vietnam, Cambodia, and Thailand. Besides, the turtle is thought to have been introduced into the Sunda Islands, Sulawesi, and Yunnan, China, through the Malay Peninsula to Sumatra, Java, and Borneo (Iverson 1992; Kuchling 1995; IUCN 2000; Koch et al. 2008; Van Dijk et al. 2012; Fritz et al. 2014; Kundu et al. 2016). Like other softshell turtles, *A. cartilaginea* exhibits a leathery shell skin, a pig-nose-like snout, four webbed feet for underwater swimming, and three powerful claws on each foot for killing and dissecting prey (Fritz et al. 2014). As a relatively large-sized freshwater turtle, the shell of an adult *A. cartilaginea* usually exceeds 40 cm in length (Van Dijk 1992) and can grow up to 83 cm (Ernst et al. 2000). Asiatic softshell turtle is listed as a vulnerable (VU) species by the International Union for Conservation of Nature (IUCN 2000), following years of overexploitation as pets and bush meat(Kundu et al. 2016). Softshell turtles vary in color and body proportions, as such morphological data is of limited use in taxonomy and systematics. The application of modern molecular techniques has improved the understanding of the taxonomy of softshell turtles, and thus focus is increasingly being directed in the generation and analysis of molecular genetics data (Fritz et al. 2014). This study aimed to determine the complete mitogenome of *A. cartilaginea* and its phylogenetic relationships with other turtle species. The findings of the present study will lay a foundation for future research and protection of *A. cartilaginea*.

We extracted muscle tissue samples from dead Asiatic softshell turtles found at the Dianchi Lake, Kunming, Yunnan, China. The samples were stored at the Museum of Kunming Institute of Zoology, Yunnan province, China (Institute coordinates: 25°4′12.87″N, 102°42′12.05″E., elev. 1900 m). Total genomic DNA was extracted from the muscle tissues using the Ezup pillar genomic DNA extraction kit (Sangon Biotech, Shanghai, China). Next-generation sequencing (NGS) of the mitochondrial genome was performed by the Personal Biotechnology Co, Ltd (Shanghai, China), as described previously (Metzker 2010). Briefly, we first constructed approximately 400 bp insert size libraries using a Whole Genome Shotgun (WGS) strategy. The libraries were sequenced using a 2 × 250 bp paired-end (PE) protocol based on the Illumina MiSeq sequencing platform. Next, the 38,596,792 reads were filtered in the raw data to generate a high quality (HQ) clean data using Adapter Removal v2 and SOAPec v2.01software. The filtered 37,868,126 reads were then used for *de novo* assembly by A5-miseq v20150522 and SPAdes v3.9.0 software. BLASTn (BLASTC v2.2.31+) in NCBI was applied to identify the contigs of mitogenome sequences. Pilon v1.18 was used to evaluate the accuracy and completeness of the genome assembly. Finally, the complete mitogenome sequence with validated annotations was submitted to GenBank with accession number MT039230.

The mitogenome of *A. cartilaginea* was to be 16,763 bp in length and encodes 37 genes, including 13 protein-coding genes (PCGs), two ribosomal RNA (12S rRNA, 16S rRNA), 22 transfer RNA genes (tRNAs), and one mitochondrial control region (CR or D-loop). The base composition is 36.2% A, 25.7% T, 26.3% C, and 11.8% G, demonstrating a bias of higher AT content (61.9%) than GC content (38.1%). The positions of all genes were predicted by the MITOS Web Server (Bernt et al. 2013). All the tRNA genes are encoded on the H-strand, except for tRNA^Gln^, tRNA^Ala^, tRNA^Asn^, tRNA^Cys^, Trna^Tyr^, tRAN^Ser(UCN)^, Trna^Glu,^ and Trna^Pro^ genes. All the PCGs are encoded on the H-strand except nad6, which is the only PCG encoded on the L-strand. Most of the PCGs begin with an ATG initiation codon; only COI starts with GTG. Besides, 11 PCGs end with complete TAA, TAG, or AGA termination codon, whereas the remaining two COIII and ND4 are terminated by incomplete TA(A) or T(AA) termination codon, respectively. Furthermore, we found an additional nucleotide that causes a translational frameshift in nad3. Like most turtles hitherto studied, the gene distribution of *A. cartilaginea* was identical to the typical vertebrate mitochondrial genome (Boore 1999).

All 13 PCGs datasets were first concatenated using PhyloSuite v1.2.2 (Zhang et al. 2020). The concatenated alignment consisted of 11,322 bp for 19 species from seven families (Chelidae, Trionychidae, Kinosternidae, Cheloniidae, Emydidae, Testudinidae, and Geoemydidae). Two side-necked turtles (family: Chelidae) were selected as outgroup. Maximum likelihood (ML) phylogenies of *A. cartilaginea* were inferred using IQ-TREE v1.6.8 (Nguyen et al. 2015) under the Edge-linked partition model for 5000 ultrafast bootstraps (Minh et al. 2013). The phylogenetic trees based on the mitogenomes indicated that *A. cartilaginea* has the closest relationship with *N. nigricans* and clusters within the clade of family *Trionychidae* (Figure 1). These findings may shed light on future investigations into the molecular evolution and conservation of softshell turtle species.

**Figure 1.**
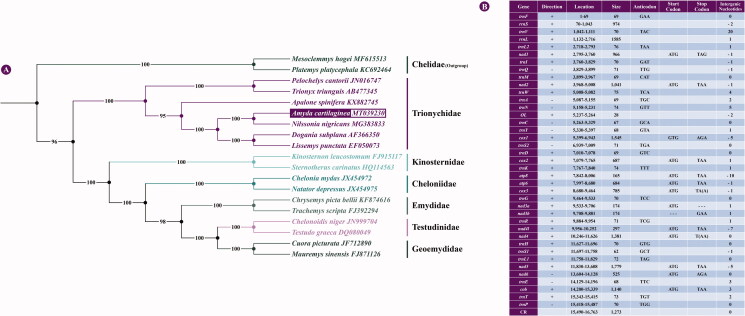
Phylogenetic tree and genomic annotation of *A. cartilaginea*. (A) Maximum-likelihood (ML) phylogenetic tree yielded by IQTREE for seven turtle families based on a concatenated alignment of 13 protein-coding genes from 19 turtle species; bootstrap values are shown in the middle of the branches connected to the nodes. (B) Annotation of the complete mitogenome of *A. cartilaginea*.

## Data Availability

The data used to support the findings of this study are available in GenBank at https://www.ncbi.nlm.nih.gov/nuccore/MT039230, reference number MT039230.
